# Mandatory requirements for pediatric drug development in the EU and the US for novel drugs—A comparative study

**DOI:** 10.3389/fmed.2022.1009432

**Published:** 2022-10-31

**Authors:** Helle Christiansen, Marie L. De Bruin, Christine E. Hallgreen

**Affiliations:** ^1^Copenhagen Centre for Regulatory Science, Department of Pharmacy, Faculty of Health and Medical Sciences, University of Copenhagen, Copenhagen, Denmark; ^2^Division of Pharmacoepidemiology and Clinical Pharmacology, Utrecht Institute for Pharmaceutical Sciences, Utrecht University, Utrecht, Netherlands

**Keywords:** EU Pediatric Regulation, Pediatric Research Equity Act, legislation, pediatric drug development, EMA, FDA

## Abstract

Mandatory pediatric legislation has been implemented in the European Union (EU) and the United States (US) to increase research and the availability of drugs for the pediatric population. Differences in the legislative framework can cause different pediatric requirements for similar indications granted for similar drugs across jurisdictions. This cross-sectional study compares the pediatric requirements for therapeutic indications granted at the time of initial approval for novel drugs approved in the two regions from 2010 to 2018. We collected the European Medicines Agency (EMA) and the US Food and Drug Administration (FDA) decisions to grant a waiver and/or to agree on a pediatric development plan and deferrals hereof at marketing authorization (MA) from publicly available documents. An agreed pediatric development plan was required for 66% (*N* = 188/285) and 63% (*N* = 134/212) of the indications granted in the EU and the US at the time of approval, respectively. Almost all (EU; 98%, US; 89%) were deferred until after MA. Based on the broad scope of the EU Pediatric Regulation, an additional 36 PIPs originated from the indications granted at MA. In the subset of indications granted for drugs approved in both the EU and the US (*N* = 232), significantly more indications resulted in an agreed pediatric development plan for one or more subsets of the pediatric population in the EU (*N* = 185) as compared to the US (*N* = 82). This was based on the exemption of orphan designated drugs in the US and the broader scope of the EU Pediatric Regulation. However, indications subject to the mandatory pediatric legislation in both regions (*N* = 131) most often had similar regulatory requirements for the inclusion of the pediatric population from the EMA and the US FDA (83%, *N* = 109). In conclusion, when comparing mandatory pediatric requirements, more pediatric development plans were agreed upon in the EU than in the US, in line with the broader mandates of the EU Pediatric Regulation. However, authorities most often had similar regulatory requirements when an indication was subject to pediatric legislation in both regions.

## Introduction

In the past, medicinal products were rarely evaluated in the pediatric population, resulting in a scarcity of drugs approved for use in the pediatric population, resulting in a high level of off-label use in this population. Since market forces have not been able to drive changes, initiatives have been implemented in several regulatory regions to support the establishment of knowledge on how to use medicinal products in the pediatric population (1). However, the European Union (EU) and the United States (US) were the first regions to introduce mandatory pediatric legislations (2, 3).

The US Pediatric Research Equity Act (PREA) made the inclusion of the pediatric population (from birth to the age of 16 years) mandatory during drug development when it came into force in December 2003 (3). It complemented the already existing voluntary Best Pharmaceuticals for Children Act (BPCA) implemented in 2002 (4) where a reward could be gained for the conduct of requested pediatric drug development. The EU Pediatric Regulation adopted in December 2006 was built upon the learnings from the US (2) and combined mandatory requirements with rewards as incentives for pediatric drug development.

Except for orphan drugs which are exempted from US PREA but not the EU Pediatric Regulation, the overall framework is quite similar across the two jurisdictions; both the US PREA and the EU Pediatric Regulation mandate submission of results from clinical studies that included the pediatric population specified in an agreed pediatric development plan (Pediatric Study Plan (PSP) in the US and Pediatric Investigation Plan (PIP) in the EU) before a marketing authorization (MA) application is considered valid unless requirements for pediatric development have been waived or deferred until after MA. Thus, if appropriate measures are not taken to include the pediatric population during the drug development of novel drugs or already approved drugs still covered by a patent or a supplementary protection certificate, entry to the market can be blocked in the EU and the US.

Besides the exemption of orphan drugs in the US PREA, also the broader scope of the mandatory EU Pediatric Regulation compared to the US PREA has been highlighted as a major difference between the two legislations, and so have the broader options/reasons for granting a waiver by US FDA compared to EMA (5). These differences can potentially lead to regional differences in the decisions on the requirements for the inclusion of the pediatric population during drug development. Such regional regulatory differences can have practical implications for applicants when running a global drug development program, which is critical to the conduct of effective, efficient, and ethical drug development for small populations, such as the pediatric population (6).

First, a difference in regulatory requirements can arise from the scope since the US PREA is restricted to the proposed indication(s) for the adult population, whereas the EU Pediatric Regulation provides a mandate for the European Medicines Agency (EMA) to require a drug development for the pediatric population for another indication *within* the condition of the proposed indication if a potential pediatric need exist (7). Therefore, a PIP can cover an indication not intended by the applicant and therefore not granted at the initial MA, but only targeted in a PIP. In this way, potential pediatric use outside the proposed adult indication cannot be ignored. Second, a difference in regulatory requirements can arise from a difference in the grounds for granting waivers. The reasons for granting a waiver are more or less the same between the EU and the US, with one exception. In the US, a waiver can be granted based on the ground that the necessary studies are impossible or highly impracticable (e.g., because the patients are geographically dispersed), but this is not the case in the EU.

In 2007, a pediatric cluster was established between the EMA and the US Food and Drug Administration (FDA) with the objective of avoiding the exposure of children to unnecessary trials and facilitating global pediatric development plans based on scientific grounds, and compatible with both agencies' legislations (8). However, consensus cannot always be reached based on different legislations, standards of care, and cultures (9). It remains to be seen if this harmonization effort can facilitate regulatory understanding leading to similar regulatory decisions between the jurisdictions (10).

To our knowledge, only one study has benchmarked the requirements for pediatric drug development between the EU and the US. This study investigated the EMA decisions for waiver applications in the EU in relation to the US FDA, showing a high similarity in decisions (13). However, the study did not give a complete overview of decisions in both regions, and it did not cover decisions for agreed pediatric development plans (PIPs or PSPs).

This study aims to provide a complete overview of the decisions by the EMA and the FDA to grant a waiver and/or to agree on a pediatric development plan (PIP or PSP) for indications granted at the initial time of MA for novel drugs approved in the EU and the US between 2010 and 2018. In addition, we analyze the concordance of regulatory decisions on the indications to be studied under a pediatric development plan for indications authorized in both regions. For this subset, we provide details on requirements for pediatric development plans for indications only subject to the EU Pediatric Regulation, but outside the scope of US PREA.

## Methods

### Study design

We performed a retrospective cross-sectional analysis of the decisions by the EMA and the FDA on the granting of waivers or the agreement of pediatric development plans (PIP or PSP) for indications at the time of the first MA for all novel drugs approved in the EU through the centralized procedure or in the US between 1 January 2010 and 31 December 2018. Novel drugs were identified using a list of New Active Substances (NAS) authorized in the US, and/or in the EU maintained by the CIRS (Center for Innovation in Regulatory Science) (14, 15) for research purposes (see Supplementary material for CIRS definition of NAS).

### Data sources

For all drugs approved in the US, the US letters and authorization information were retrieved from the FDA website, FDA's CDER (16) or CBER (17). For drugs approved in the EU, EPARs (European Public Assessment Reports) were retrieved from the EMA website and authorization information was collected from the so-called “download list” of all EPARs for human and veterinary medicines (18). The EMA decision number valid at the time of MA was identified using the EPAR section “1.1.2. Information on pediatric requirements”. This number (P/XXXX/YEAR, e.g., P/0297/2013 for Alirocumab) was used to identify the EMA decision on the agreement of pediatric investigation plans and the granting of deferrals and waivers^1^ *via* a google search. If the decision could not be found, the information was requested through the EMA access-to-documents request (19).

### Data collection

For each product, we extracted the approval date, therapeutic area [Anatomical Therapeutic Chemical (ATC) Classification based on international non-proprietary name (INN)], and orphan status in the respective region. The ATC classification was used as a starting point to match identical drugs approved in both regions, followed by manual quality checks, e.g., to assign drug pairs for further analysis in case of multiple potential matches.

For each unique ATC, the EPARs and the US letters were scrutinized to collect all indications granted at initial MA (adult and pediatric) to create the study unit of drug-indication (from now on just called indications). In addition, all EMA decisions on waivers or agreed PIPs were scrutinized to collect additional indications only targeted in a PIP (from now on referred to as “indications only targeted in a PIP”). All indications were recorded at the level of condition or disease (depending on the details in the documents) using the Medical Dictionary for Regulatory Activities (MedDRA) Preferred Terms (PTs) (20).

For each authorized indication or indication only targeted in a PIP, the corresponding decisions by the EMA and the FDA on granting a waiver or agreement on a pediatric development plan (PIP or PSP) were collected (from now on “requirements for a pediatric development plan”). The requirements for a pediatric development plan were categorized as either a “full waiver” (a waiver covering all subsets of the pediatric population), a “partial pediatric development plan” (an agreed pediatric development plan (PIP or PSP) with a waiver for one or more subsets of the pediatric population) or a “full pediatric development plan” (an agreed pediatric development plan (PIP or PSP) for the entire pediatric population). Information on deferral for one or more subsets of a partial or full pediatric development plan was also collected, as were the reasons for granting a waiver. The pediatric subgroups (adolescent, children, toddler and infants, and term newborn) were defined by the International Conference on Harmonization (ICH) Topic E 11, 2001 (21).

### Data analysis

For each region, we reported on granted waivers and agreed pediatric development plans (full or partial) with deferrals hereof in absolute numbers and percentages for all approved indications, and stratified by therapeutic area. Therapeutic areas were defined according to the primary System Organ Class (SOC) of the MedDRA (20) covered by each indication. Further, we reported on the reasons for granting waivers in each region. In addition, we provided an overview of the concordance between the decisions by the EMA and FDA on granting waivers and/or agreement of pediatric development plans for indications granted for drugs approved in both the EU and the US. Further, for each pediatric subgroup, we tested if there was a difference in requirements for pediatric development in EU and US, using χ^2^ test of independence. All calculations were performed using statistical software R, version 3.6.0 (2019-04-26) (22).

## Results

### Characteristics of study sample

**Figure 1:**
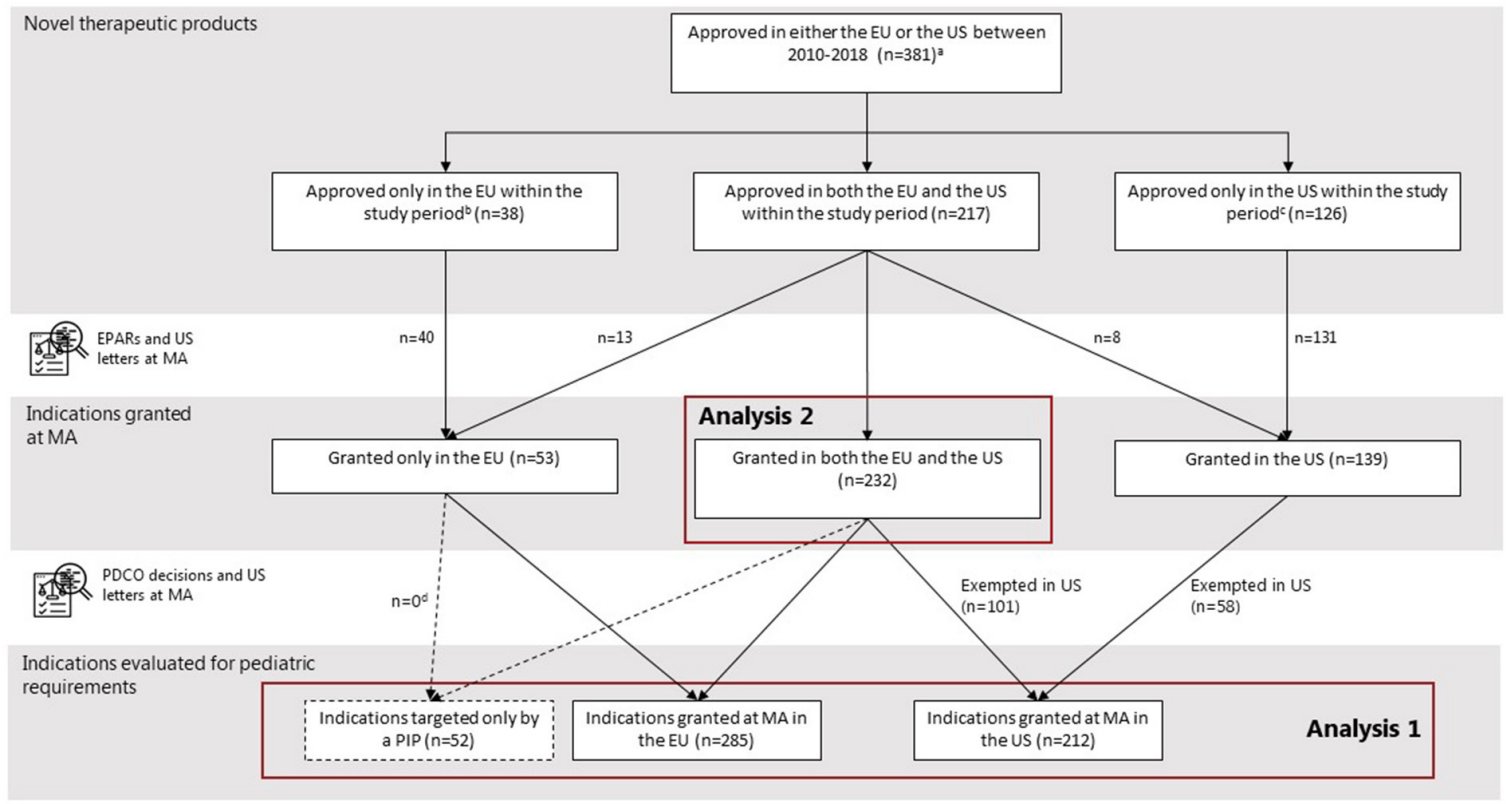
Selection of study cohort. ^a^Two NAS were excluded because they were not defined as a NAS by the EMA and the US. ^b^In the US, some drugs were approved before (*N* = 12) or after the (*N* = 4) study period, not considered novel at initial MA (*N* = 5), or not approved (*N* = 17). ^c^In the EU, some drugs were approved before (*N* = 18) or after (*N* = 22) study period, not considered novel at initial MA (*N* = 8), not approved through the centralized procedure (*N* = 4), or not approved (*N* = 74). ^d^None of the indications targeted only by a PIP originated from the indications granted only in the EU at time of MA.

From 2010 to 2018, 255 drugs were approved in the EU through the centralized procedure as novel therapeutics, comprising 285 indications at MA (Figure 1). In the same period, the FDA approved 343 drugs as novel therapeutics, comprising 371 indications. All 285 indications granted in the EU were subject to the EU Pediatric Regulation. In addition, we observed additional 52 indications only targeted in a PIP originating from the approved indications at MA. In the US, only 212 indications were subject to the US PREA since 159 indications were granted an orphan drug designation exempting them from mandatory pediatric requirements.

### Mandatory pediatric requirements in the EU and the US

The majority of the indications granted at MA and being subject to the EU Pediatric Regulation or the US PREA (EU: 66%, 188/285, US: 63%, 134/212), had a partial (EU; *N* = 114, US; *N* = 100) or full (EU; *N* = 74, US; *N* = 34) pediatric development plan (see Table 1). However, almost all (EU; 98%, *N* = 185/188, US; 89%, *N* = 119/134) were deferred for at least one measure until after MA. In the US, pediatric development plans had been completed at MA for 15 indications covering 15 drugs (for details see Supplementary Table S2). In the EU, this was the case for three indications granted for three different drugs.

**Table 1:** Granted waivers and agreed pediatric development plans (PIP or PSP) for novel drug indications granted at MA between 2010 and 2018 - US (*N* = 371) and EU (*N* = 285).

	**US**		**EU**			
			**Indications granted**		**Indications only targeted**	
			**at MA**		**in a PIP**	
	**No**.	**(%)**	**No**.	**(%)**	**No**.	**(%)**
Number of indications evaluated for pediatric requirements	212^a^	(100%)	285	(100%)	52	(100%)
Number of indications granted a full waiver	78	(37%)	97	(34%)	16	(31%)
Number of indications with a full pediatric development plan	34	(16%)	74	(26%)	9	(17%)
- Of which deferred until after MA^b^	27	(13%)	72	(25%)	9	(17%)
Number of indications with a partial pediatric development plan	100	(47%)	114	(40%)	27	(52%)
- Of which deferred until after MA^b^	92	(43%)	113	(40%)	27	(52%)
Age categories covered by partial pediatric development plans						
- Adolescents (12–18 years^c^)^d^	100	(47%)	114	(40%)	26	(50%)
- Children (2–11 years)^d^	31	(15%)	44	(15%)	20	(38%)
- Toddlers and infants (27 days-23 months)^d^	7	(3%)	7	(2%)	3	(6%)
- Term newborn (0–26 days)	0	(0%)	0	(0%)	1^e^	(2%)

^a^The numbers differ from the actual indications granted at the initial MA (given in the header) since 159 indications were exempted in the US due to an orphan drug designation.

^b^Those not deferred had a compliance check at MA. In the US, a statement of correct indications for population or fulfillment of pediatric requirements was made.

^c^12–17 years in the US.

^d^Waiver can include one or more subsets of the pediatric population.

^e^PIP only agreed for term newborns for the indication of neonatal seizures.

Pediatric requirements were not always mandated for the 52 indications only targeted in a PIP as 16 were granted a waiver. None of the 36 agreed pediatric development plans (full: *N* = 9, partial: *N* = 27) had been completed at the time of MA, all were granted a deferral.

For all indication, partial pediatric development plans most often only included adolescents and to some extend children, and the youngest age groups were rarely covered. Only one indication (neonatal seizure) had a pediatric plan covering term newborns (neonates) (see Table 1).

#### Waiver reasons

In the US, most waivers (87%, full waivers: *N* = 72/78, waivers granted for a subset of the pediatric population: *N* = 83/100) were granted because necessary studies would be impossible or highly impracticable (Table 2). Whereas in the EU, waivers most often (65%, full waivers *N* = 43/133 and waivers granted for a subset of the pediatric population *N* = 122/141) were justified based on no significant therapeutic benefit in the pediatric population or the presence of a low number of pediatric patients for the given indication. In both regions, only few waivers were granted based on safety issues.

**Table 2:** Reasons for granting a full waiver or a waiver for one or more subgroups of the pediatric population for indications evaluated by the EMA PDCO (N = 337) or the US FDA (N = 212).

	**EU**		**US**	
	**No**.	**(%)**	**No**.	**(%)**
**Full waiver**				
Number of indications granted a full waiver	113^a^	(100%)	78^a^	(100%)
- Class waiver	58	(51%)	NA	NA
- Product-specific	55	(49%)	NA	NA
The necessary studies are impossible or highly impracticable	NA	NA	72	(92%)
Ineffective or unsafe	6	(5%)	2	(3%)
No significant therapeutic benefit OR a low number of pediatric patients	43	(38%)	2	(3%)
- The condition or disease for which the specific medicinal product or class is intended occurs only in the adult population	28	(25%)	NA	NA
- The specific medicinal product does not represent a significant therapeutic benefit over existing treatments for pediatric patients	15	(13%)	NA	NA
No reason provided^b^	64	(57%)	2	(3%)
**Waiver for one or more subgroups of the pediatric population**				
Number of indications with an agreed PIP for only a subset of the pediatric population	141^c^	(100%)	100^c^	(100%)
The necessary studies are impossible or highly impracticable	NA	NA	83	(83%)
Ineffective or unsafe	17	(12%)	5	(5%)
No significant therapeutic benefit OR a low number of pediatric patients	122	(87%)	9	(9%)
- The condition or disease for which the specific medicinal product or class is intended occurs only in the adult population	46	(33%)	NA	NA
- The specific medicinal product does not represent a significant therapeutic benefit over existing treatments for pediatric patients	76	(54%)	NA	NA
No reason provided	2	(1%)	3	(3%)

Percentages are calculated from the number of indications with a full waiver or the number of indications with a waiver for one or more subgroups of the pediatric population.

^a^For 34 indications a waiver was granted in both regions.

^b^The most common ground for not providing a reason in the EU was indications for medicines covered by a class waiver (N = 58). Class waivers are granted to medicines that are likely unsafe or ineffective in children, lack benefit for children or are for diseases and conditions that only affect the adult population.

^c^For 57 indications a waiver was granted in both regions for one or more subgroups.

#### Therapeutic areas of waivers and pediatric development plans

**Figure 2:**
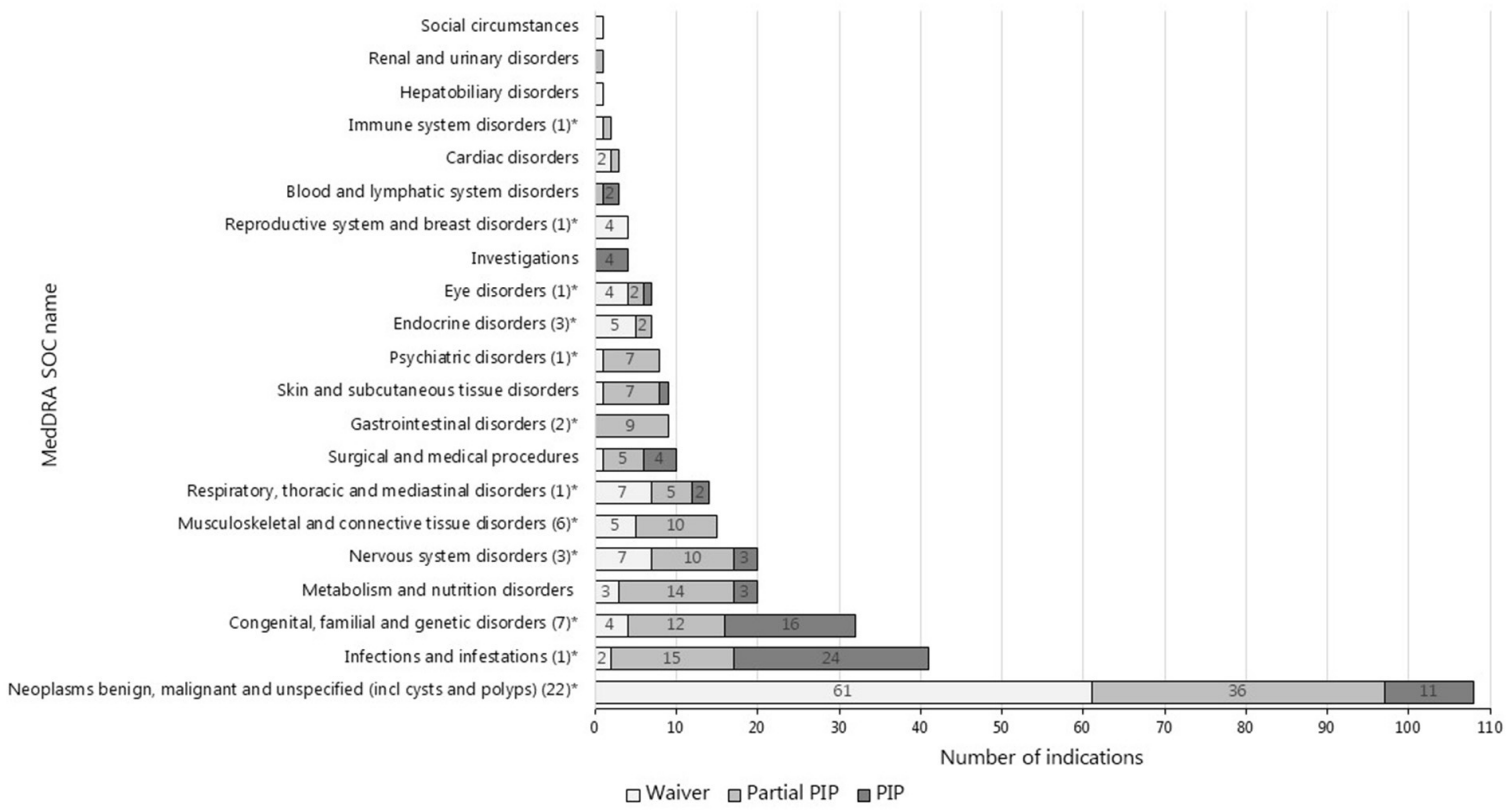
Therapeutic areas of indications evaluated for pediatric requirements in the EU (*N* = 337^#^). ^#^This is the total number of indications evaluated for pediatric requirements in the EU arising from the indications approved at MA (*N* = 285) and the indications only targeted in a PIP (*N* = 52). *The number of indications targeted only by the PIP is provided in the brackets.

**Figure 3:**
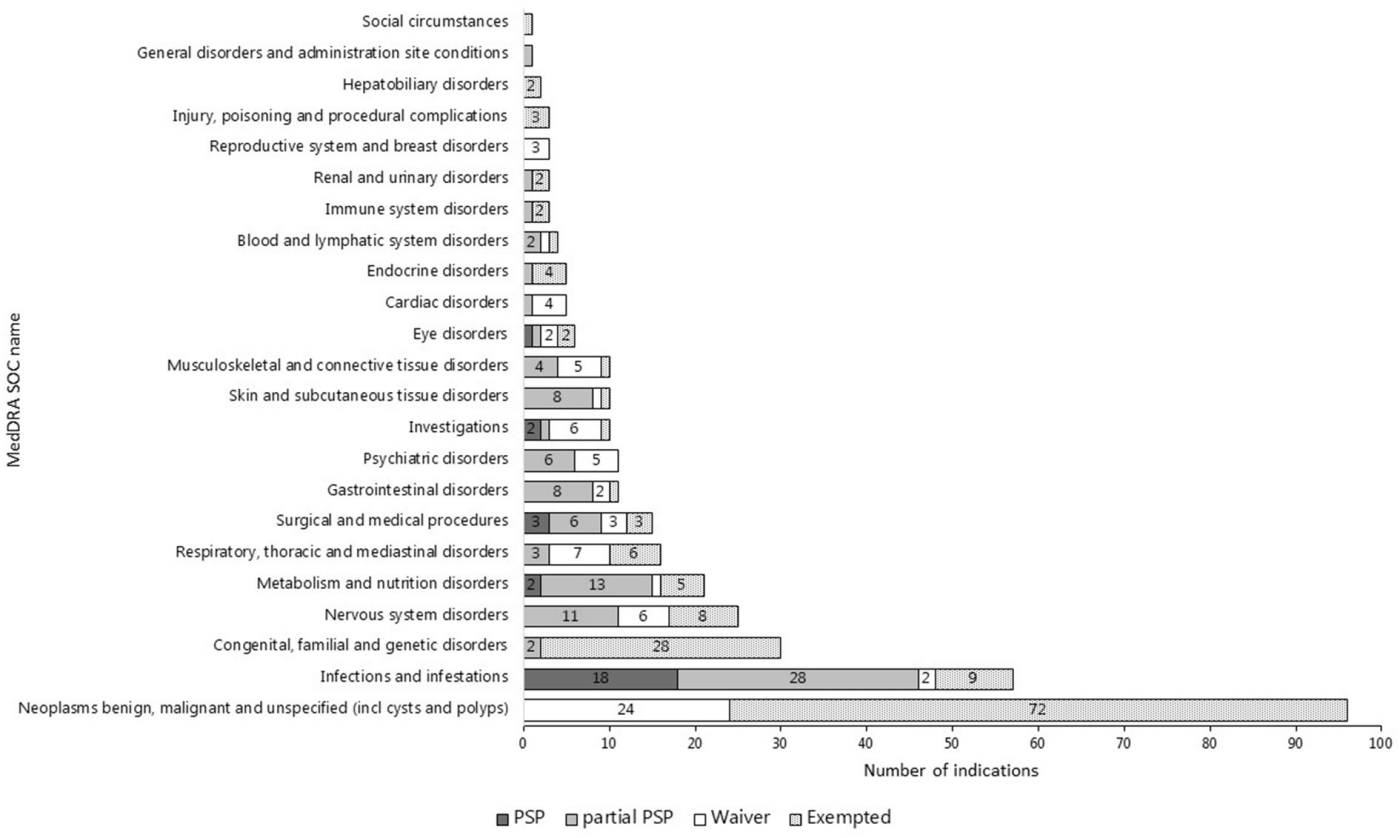
Therapeutic areas of indications evaluated for or exempted from pediatric requirements in the US (*N* = 371).

The top three most common therapeutic areas evaluated for pediatric requirements in both jurisdictions consisted of cancer, infections/infestations, and inherited disorders (Figures 2, 3). However, in the US, a PSP was agreed for only a minority of the indications within the field of inherited disorders and cancer as these indications often were granted a waiver. In the EU, a bit less than half of the indications evaluated for pediatric requirements within the field of cancer were waived, however, most indications within inherited disorders and infections/infestations had an agreed PIP with a development plan for at least a subset of the pediatric population, but even more frequently for the entire population.

The indications only targeted in a PIP most often also covered the therapeutic area of cancer (*N* = 22) and inherited disorders (*N* = 7), but also musculoskeletal and connective tissue disorders (*N* = 6) were covered (see Figure 2).

### Differences in regulatory decisions for indications *authorized* in both the EU and the US

**Figure 4:**
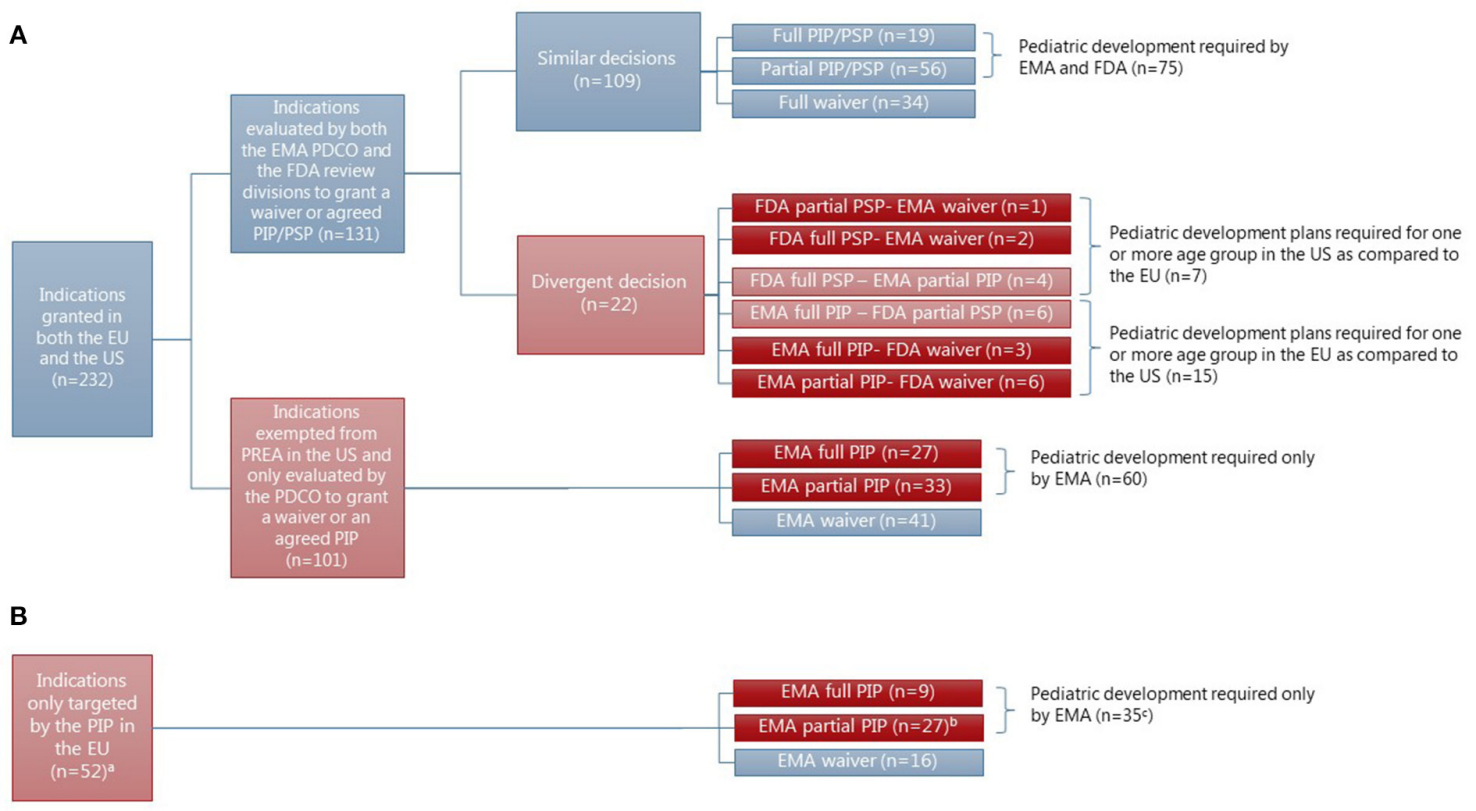
Concordance in the granting of waivers or agreement of PIP/PSP for indications granted at MA in both the EU and the US (*N* = 232) for drugs approved in both regions (*N* = 217). ^a^Two indications were granted approval at MA by the FDA but not the EMA, however, they were evaluated by the EMA for granting of waiver or agreement of PIP as indications only targeted in a PIP in the EU. ^b^One indication (for linaclotide-constipation) was approved in the US at MA and a pediatric development plan was agreed upon for adolescents in both the EU and the US. ^c^The number does not fit since one indication (constipation) had an agreed pediatric development plan in the US as well. **(A)** Indication granted at the time of initial approval of novel drugs in the EU and the US. **(B)** Indication only targeted in a PIP.

In the subset of indications granted at MA for drugs approved in both the EU and the US and the indications only targeted in a PIP originating hereof (*N* = 284) (see Figures 4A,B), the statistical analysis showed a significant difference between the pediatric requirements mandated in the EU and the US for all the pediatric subgroups (adolescents: X-squared = 69.052, df =1, *p* < 2.2e-16, children: X-squared = 55.476, df =1, *p* = 9.459e-14, toddlers and infants: X-squared = 22.095, df =1, *p* = 2.594e-06, term newborns: X-squared = 20.082, df =1, *p* = 7.419e-06) (see Supplementary Table S6).

The majority of differences were based on indications with an orphan drug designation in the US, thereby exempting them from US PREA (*N* = 101). For 60 of these indications (see Figure 4A), a pediatric development plan was required in the EU; either for the entire pediatric population (*N* = 27), adolescents and children (*N* = 20), or only adolescents (*N* = 13). The therapeutic areas were most often covered by an agreed full or partial PIP for cancer (*N* = 25) and inherited disorders (*N* = 17). However, more than half of the indications covering cancer (*N* = 29) were granted a waiver in the EU, resulting in no pediatric development plan in either of the regions (see Supplementary Table S3). No waivers were granted in the EU for indications within the area of inherited disorders.

The remaining differences emerged from indications only targeted in a PIP in the EU, with a pediatric development plan agreed for 35 indications for at least one subset of the pediatric population as compared to the US (see Figure 4B). Two of the indications with an agreed pediatric development plan in the EU had also been evaluated for pediatric requirements in the US, but only one resulted in a pediatric development plan.

### Concordance in regulatory decisions for indications *evaluated* for pediatric requirements in both the EU and the US

In the subset of indications granted at the time of initial approval of novel drugs in the EU and the US and subject to the mandatory pediatric legislations in both the EU and the US (*N* = 131), no statistically significant difference was found between pediatric requirements mandated in the EU and the US for any of the four pediatric subgroups (see Supplementary Table S7). For these indications, the EMA and the FDA made a similar decision for the vast majority [83%, *N* = 109 (see Figure 4A)]. Even decisions on deferrals and the included age groups of a partially agreed pediatric development plan were most often similar. Of the 19 indications with an agreed pediatric development plan for the entire pediatric population, 14 were granted a deferral in both regions and four were granted only in the EU. For one indication (hemophilia A), the agreed pediatric development plan had been completed at MA in both the EU and the US (Supplementary Table S2). Of the agreed partial pediatric development plans (*N* = 56) in both the EU and the US, the included age groups only differed for four indications covering children (waiver granted in the US: *N* = 2 or EU: *N* = 1) or toddlers and infants (waiver granted in the US: *N* = 1). However, a divergent decision was made by the EMA and the FDA for 22 indications (17%) (see Figure 4), most often resulting in a pediatric development plan agreed for more subsets of the pediatric population in the EU as compared to the US (*N* = 15) (see Figure 4, for details, see Supplementary Table S5).

## Discussion

Global drug development is necessary to avoid duplication of clinical trials and decrease the time to patient access, especially when developing drugs for small populations such as the pediatric population. Global development activities depend very much on an ambition to harmonize regulatory requirements around the world to enable an aligned development strategy.

This study provides an analysis of the degree of differences and similarities in regulatory requirements for pediatric drug development based on the mandatory pediatric legislations in the EU and the US for novel drugs approved in both region. Our study shows an overall significant difference in the pediatric requirements mandated by the EMA and the FDA for indications granted at MA that can be attributed to the differences between the EU Pediatric Regulation and the US PREA.

The differences seen in the regulatory requirements mainly arise from the exemption of orphan drug designated indications, which constitute a little less than half of the indications granted in both the US and the EU, most often covering cancer diseases and to a smaller extent inherited disorders. In general, it has been shown that the US FDA grants more orphan drug designations as compared to the EMA (23) and therefore, the exemption could have a rather large impact. However, a recent study with a similar study sample, found only a few discrepancies between the guidance for pediatric use in the prescription information (24), suggesting that the impact of the observed differences in requirements on the regulatory output is rather small. There could be several reasons for this. First, the pediatric drug development for orphan drugs in the US could be driven by other regulatory policies such as the US BPCA or the orphan drug legislation. The orphan drug legislation provides incentives to develop drugs to prevent, diagnose, or treat rare diseases and conditions, including in pediatric patients. The US BPCA has been shown as the predominant policy contributing to pediatric drug development for cancer drugs in the US (25). This development is important as many of the drugs exempted by the US PREA have been shown to have a mechanism of action warranting pediatric development plans (26). Second, a spillover effect from the regulatory region with the strongest mandate could occur, however, previous studies have shown only a small number of medicines for pediatric populations arising based on regulatory actions in other regions (27, 28). Lastly, the progress of the pediatric development plans in the EU has been questioned in general and the impact of differences in regulatory requirements could also be reduced if the agreed pediatric development plans are never completed.

Recent numbers suggest that we will continue to see that orphan drug designation compromises around half of the drugs approved in the US (29). However, in the future the difference in mandated pediatric development plans could be reduced as an amendment to the US PREA became effective in August 2020, allowing regulators to mandate a PSP for adult cancer drugs if directed at a molecular target also relevant to the growth or progression of pediatric cancer (30). This amendment also includes required studies for cancer indications with an orphan drug designation.

Our study is the first to suggest a method to investigate the outcome of the broad mandate by the EMA PDCO to require a pediatric drug development that does not only follow the proposed indication by the MA applicant. This is done by tracing the indications only targeted in a PIP in the EU, thereby possibly agreeing to an indication different from the proposed indication, but still within the condition hereof. Using this method, we demonstrate that the EMA PDCO uses this broad mandate to a certain extent and that it contributes to the difference in pediatric requirements mandated by the EMA and the FDA for indications granted at MA. However, the voluntary conduct of requested pediatric studies through the US BPCA is intended for development outside the proposed indication and could reduce the differences in practice. Unfortunately, such information is not released until the development has been completed and is therefore not publicly available at the time our study was conducted, why we cannot conclude on its contribution. A recent publication showed that after ~5 years, the potential pediatric use outside an adult indication was rarely included in either the EU or US prescription information (29). This can either be seen as a failure to complete the agreed pediatric development or as a symptom of the complex and long duration of pediatric drug development always being a step behind adult development (31).

Our study also shows an alignment in the EMA and the FDA decisions on pediatric requirements for the indications subject to the mandatory pediatric legislations in both regions. This suggests, that even though a broader basis exists for granting waivers in the US than in the EU, it does not result in any significant differences when pediatric development is required in the two regions. Our findings support previously published findings of Egger et al. (13) who found a high concordance in waiver decisions between the EMA PDCO and FDA. Both agencies are involved in ongoing efforts to harmonize regulatory decisions regarding requirements for pediatric development plans such as the pediatric cluster meetings and guidelines on transparency regarding the advice and agreements of pediatric studies with other regulatory authorities (32, 33). While we cannot claim that the high concordance in decision-making on pediatric development plans observed in this study is a result of these harmonization efforts, their continued use is encouraged.

The results should be interpreted within the limitations of this study. First, the study is a snapshot in time, showing the EMA and the FDA decisions on waivers and pediatric development plans at MA. However, the agreed pediatric development plans are dynamic, with possible modifications after the initial agreement and MA. Second, we did not investigate if the applications for waivers or agreed pediatric development plans were similar in the EU or the US. Instead, we assumed that the basis for the EMA and the FDA decision was similar if similar indications were approved at MA. On the same basis, our study might overestimate the indications only targeted in a PIP, as these could have been derived from earlier proposed indications at the time of application. Third, this study only investigates the mandatory requirements for pediatric development plans without including the voluntary Written Requests issued as part of the US BPCA and does not provide an overview of the entire pediatric development plans taken on by companies in response to pediatric legislations in the US. The potential differences seen from the mandatory legislations could be diminished by a request for pediatric studies through a Written Request (WR) using the US BPCA.

In the subset of indications, where the EU and the US regulators evaluated pediatric requirements on the same grounds, the similarity of the pediatric programs required in both regions remains to be explored. The type of information required in the submission of pediatric development plans is similar (34), but the actual plans with regard to e.g., the number, purpose, design, duration, and timing of required pediatric studies can still differ between regions.

In conclusion, when comparing purely compulsory requirements for pediatric studies for drugs approved in both the EU and the US, a larger number of pediatric development plans were agreed upon in the EU, in line with the broader mandates of the EU Pediatric Regulation. When both regulatory authorities evaluated an indication for requirements for pediatric development plans, they most often made similar decisions regarding waivers and pediatric development plans, and deferrals hereof.

## Data availability statement

The original contributions presented in the study are included in the article/Supplementary material, further inquiries can be directed to the corresponding author.

## Author contributions

HC, MD, and CH wrote the manuscript and designed the research. HC and CH performed the research. HC analyzed the data. All authors contributed to the article and approved the submitted version.

## Funding

HC is a Ph.D. student at CORS and project was funded by a grant from Lundbeck A/S to the Copenhagen Centre for Regulatory Science (CORS) at the University of Copenhagen. CORS is a cross-faculty university-anchored institution involving various public (Danish Medicines Agency, Copenhagen University) and private stakeholders (Novo Nordisk, Lundbeck, Ferring pharmaceuticals, LEO pharma) as well as patient organizations (Rare Diseases Denmark). The center is purely devoted to the scientific aspects of the regulatory field and with a patient-oriented focus. The research is not a company-specific product or directly company-related.

## Conflict of interest

The authors declare that the research was conducted in the absence of any commercial or financial relationships that could be construed as a potential conflict of interest.

## Publisher's note

All claims expressed in this article are solely those of the authors and do not necessarily represent those of their affiliated organizations, or those of the publisher, the editors and the reviewers. Any product that may be evaluated in this article, or claim that may be made by its manufacturer, is not guaranteed or endorsed by the publisher.

## Footnotes

^1^Opinion of the Pediatric Committee on the agreement of a Pediatric Investigation Plan and a deferral and a waiver.
