# Supplementary material for: Mandatory requirements for pediatric drug development in the EU and the US for novel drugs—A comparative study
Source: Front Med (Lausanne). 2022 Oct 31;9:1009432. doi: 10.3389/fmed.2022.1009432 (PMC9659631; doi:10.3389/fmed.2022.1009432)
Supplement: Supplementary file 3 [file Data_Sheet_3.docx]

# **Appendix A: CIRS definition of New Active Substances (NAS)**

CIRS defines a NAS as a chemical, biological, biotechnology, or radiopharmaceutical substance that has not been previously available for therapeutic use in humans and is destined to be made available as a ‘prescription only medicine’, to be used for the cure, alleviation, treatment, prevention or in vivo diagnosis of diseases in humans. The term NAS also includes:

• Isomers, mixtures of isomers, complexes, derivatives or salts of a chemical substance previously available as a medicinal product but differing in properties with regard to safety and efficacy from that substance previously available

• Biological or biotech substances previously available as a medicinal product, but differing in molecular structure through changes to the nature of source material or manufacturing process and which will require clinical investigation

• Radiopharmaceutical substances that are a radionuclide or a ligand not previously available as a medicinal product. Alternatively, the coupling mechanism linking the molecule and the radionuclide has not been previously available

The term NAS excludes:

• Vaccines

• Biosimilars

• Any other application, where new clinical data were submitted

• Generic applications

• Those applications where a completely new dossier was submitted from a new company for the same indications as already approved for another company

• Applications for a new or additional name, or a change of name, for an existing compound (i.e., a ‘cloned’ application)

**Table S1. Characteristics of the novel therapeutics approved between 2010 and 2018 in the EU and the US (EU: n=255, US: n=343)**

|  | **EU** | | **US** | |
| --- | --- | --- | --- | --- |
|  | **no.** | **%^a^** | **no.** | **%^a^** |
| Total number of products^a^ | 255 | 100% | 343 | 100% |
| - *Number of products with an orphan drug designation for at least one indication^b^* | *62^c^* | *24%* | *153^c^* | *45%* |
| Median number of indications granted per product at MA | 1 | range 1-4 | 1 | range 1-4 |
| Number of products approved in both region | 217 | 85% | 217 | 63% |
| - *First region of approval* | *39* |  | *178* |  |
| - *Median lag time in days between approval in one region to the approval in the other region* | *91* |  | *186* |  |
| Approval year |  |  |  |  |
| 2010 | 13 | 5% | 19 | 6% |
| 2011 | 24 | 9% | 32 | 9% |
| 2012 | 21 | 8% | 37 | 11% |
| 2013 | 29 | 11% | 29 | 8% |
| 2014 | 31 | 12% | 44 | 13% |
| 2015 | 39 | 15% | 50 | 15% |
| 2016 | 28 | 11% | 23 | 7% |
| 2017 | 30 | 12% | 50 | 15% |
| 2018 | 40 | 16% | 59 | 17% |

^a^Percentages calculated from the total number of products. ^b^An orphan drug designation is granted per indication ^c^For 50 products, at least one orphan drug designation was granted in both regions. Of which 217 products were authorized in both the EU and the US, see also figure 1.

**Table S2. Products where the relevant pediatric drug development had been completed for indications granted at MA in the EU (n=3) and the US (n=15).**

| **Product name** | **Generic name** | **Indications granted at MA** | **Pediatric requirements at MA** |
| --- | --- | --- | --- |
|  |  |  |  |
| **EU** | | | |
| Strimvelis | Autologous CD34+ enriched cell fraction | Severe combined immunodeficiency due to adenosine deaminase deficiency (ADA-SCID) | Full PIP |
| Jivi | Damoctocog alfa pegol | Haemophilia A (congenital Factor VIII deficiency) | Full PIP |
| Luxturna | Voretigene neparvovec | Inherited retinal dystrophy | PIP covering adolescent |
| **US** | | | |
| Raxibacumab | Raxibacumab | Inhalational anthrax (rare acute systemic infection) | Full PIP |
| Anthim | Obiltoxaximab | Inhalational anthrax (rare acute systemic infection) | Full PIP |
| Nuwiq | Antihemophilic Factor (Recombinant), rAHF | Haemophilia A (congenital Factor VIII deficiency) | Full PIP |
| Novoeight | Antihemophilic Factor (Recombinant) | Haemophilia A (congenital Factor VIII deficiency) | Full PIP |
| Jivi | antihemophilic factor [recombinant], PEGylated-aucl | Haemophilia A (congenital Factor VIII deficiency) | Full PIP |
| Afstyla | Antihemophilic Factor (Recombinant), Single Chain | Haemophilia A (congenital Factor VIII deficiency) | Full PIP |
| Rebinyn | GlycoPEGylated rFIX | Haemophilia A (congenital Factor VIII deficiency) | Full PIP |
| Xepi | Ozenoxacin | Impetigo | PIP covering adolescent and children |
| Ella | Ulipristal acetate | Contraception | PIP covering adolescent |
| Natazia | Estradiol valerate | Prevention of pregnancy | PIP covering adolescent |
| Annovera | segesterone acetate and ethinyl estradiol vaginal system | Prevention of pregnancy | PIP covering adolescent |
| Seysara | Sarecycline | Acne vulgaris | PIP covering adolescent |
| Cinqair | Reslizumab | Asthma | PIP covering adolescent |
| Xtoro | Finafloxacin | Acute otitis externa (AOE) | PIP covering adolescent and children |
| Lastacaft | Alcaftadine | Itching associated with allergic conjunctivitis. | PIP covering adolescent and children |

**Table S3. Therapeutic areas of indications evaluated for pediatric requirements in the EU (n=101) but having an orphan drug designation in the US split into the evaluation by the PDCO.**

| SOC names | All indications | Waiver granted in the EU | | PIP agreed for one or more subsets in the EU | |
| --- | --- | --- | --- | --- | --- |
|  | no. | no. | %^b^ | no. | %^b^ |
| **Number of indications** | **101** | **41** |  | **60** |  |
| Neoplasms benign, malignant and unspecified (incl cysts and polyps) | 54 | 29 | 54% | 25 | 46% |
| Infections and infestations | 3 | 0 | - | 3 | 100% |
| Congenital, familial and genetic disorders | 17 | 0 | - | 17 | 100% |
| Metabolism and nutrition disorders | 1 | 0 | - | 1 | 100% |
| Nervous system disorders | 3 | 2 | 67% | 1 | 33% |
| Musculoskeletal and connective tissue disorders | 1 | 1 | 100% | 0 | - |
| Respiratory, thoracic and mediastinal disorders | 5 | 2 | 40% | 3 | 60% |
| Surgical and medical procedures | 2 | 0 | - | 2 | 100% |
| Skin and subcutaneous tissue disorders | 1 | 0 | - | 1 | 100% |
| Gastrointestinal disorders | 1 | 0 | - | 1 | 100% |
| Eye disorders | 2 | 0 | - | 2 | 100% |
| Endocrine disorders | 2 | 2 | 100% | 0 | - |
| Immune system disorders | 1 | 1 | 100% | 0 | - |
| Vascular disorders | 6 | 2 | 33% | 4 | 67% |
| Hepatobiliary disorders | 1 | 1 | 100% | - | - |
| Social circumstances | 1 | 1 | 100% | - | - |

^b^Percentages are calculated from all the indications evaluated for a pediatric requirement within a therapeutic area and ordered after all indications, PIP agreed and waiver granted.

**Table S4. Indications evaluated for a pediatric requirement in the EU but exempted in the US PREA (n=101).**

|  | Indications with an orphan drug designation in the US also approved in the EU | |
| --- | --- | --- |
|  | no. | %^a^ |
| Number of indications | 101 | 100% |
| Number of indications granted a full waiver | 41 | 41% |
| Number of indications with a full pediatric development plan (deferred) | 27 (27) | 27% |
| Number of indications with a partial pediatric development plan (deferred)^a^ | 33 (32) | 33% |
| - *Included adolescents (12-18 years^b^)^c^* | *33* | *100%* |
| - *Included children (2-11 years) ^c^* | *20* | *60%* |
| - *Included toddlers and infants (27 days-23 months) ^c^* | *2* | *6%* |
| - *Included term newborn (0-26 days)* | *0* | *0%* |

^a^Those not deferred had a compliance check at MA. In the US, a statement of correct indications for population or fulfillment of pediatric requirements was made. ^b^12-17 years in the US. ^c^Waiver can include one or more subsets of the pediatric population.

**Table S5: Pediatric subgroups and waiver reasons covered by EMA and FDA divergent decisions (n=22).**

| Active substance | Indication (MedDRA preferred term) | EMA decision | FDA decision | Pediatric age groups covered by the difference | Waiver reason in the EU | Waiver reason in the US |
| --- | --- | --- | --- | --- | --- | --- |
| Pediatric drug development for more subgroups in the EU (n=15) | | | | | | |
| Cangrelor | Cardiovascular event prophylaxis | Full PIP | Full waiver | All | - | Not feasible^d^ |
| Tilmanocept | Lymphatic mapping (diagnostic) | Full PIP | Full waiver | All | - | Not feasible^d^ |
| Fluciclovine (18F) | Prostatic specific antigen increased | Full PIP | Full waiver | All | - | Not feasible^d^ |
| Sacubitril / valsartan | Cardiac failure chronic | Partial PIP | Full waiver | Adolescents, children, infants, and toddlers | Safety concerns^a^ | Not feasible^d^ |
| Naloxegol | Constipation | Partial PIP | Full waiver | Adolescents and children | Safety concerns^a^ | Not feasible^d^ |
| Apremilast | Psoriatic arthropathy | Partial PIP | Full waiver | Adolescents and children | Disease or condition do not occur in pediatric population^b^ | Not feasible^d^ |
| Baricitinib | Rheumatoid arthritis | Partial PIP | Full waiver | Adolescents and children | Disease or condition do not occur in pediatric population^b^ | Not feasible^d^ |
| Vorapaxar | Cardiovascular event prophylaxis | Partial PIP | Full waiver | Adolescents | Safety concerns^a^ | Not feasible^d^ |
| Ocrelizumab | Primary progressive multiple sclerosis | Partial PIP | Full waiver | Adolescents | No significant therapeutic benefit^c^ | Not feasible^d^ |
| Doravirine | HIV infection | Full PIP | Partial PSP | Term newborn | - | No significant therapeutic benefit or the disease or condition does not occur in the pediatric population^e^ |
| Retigabine | Partial seizures | Full PIP | Partial PSP | Term newborn | - | Not feasible^d^ |
| Perampanel | Generalised tonic-clonic seizure | Full PIP | Partial PSP | Term newborn | - | Not feasible^d^ |
| Brivaracetam | Epilepsy | Full PIP | Partial PSP | Term newborn | - | Not feasible^d^ |
| Fidaxomicin | Clostridium difficile colitis | Full PIP | Partial PSP | Infants and toddlers, and term newborn | - | Not feasible^d^ |
| Bezlotoxumab | Clostridium difficile infection | Full PIP | Partial PSP | Infants and toddlers, and term newborn | - | Not feasible^d^ |
| Pediatric drug development for more subgroups in the US (n=7) | | | | | | |
| Netupitant / palonosetron | Prophylaxis of nausea and vomiting | Full waiver | Full PSP | All | No significant therapeutic benefit^c^ | - |
| Ocriplasmin | Vitreous adhesions | Full waiver | Full PSP | All | Disease or condition do not occur in pediatric population^b^ | - |
| Eravacycline | Abdominal infection | Full waiver | Partial PSP | Adolescents | Safety concerns^a^ | Safety concerns^a^ |
| Bictegravir, emtricitabine, tenofovir alafenamide, fumarate | HIV infection | Partial PIP | Full PSP | Term newborn | No significant therapeutic benefit^c^ | - |
| Sodium zirconium cyclosilicate | Hyperkalaemia | Partial PIP | Full PSP | Term newborn | No significant therapeutic benefit^c^ | - |
| Rolapitant | Prophylaxis of nausea and vomiting | Partial PIP | Full PSP | Infants and toddlers and term newborn | Safety concerns^a^ | - |
| Rilpivirine hydrochloride | HIV infection | Partial PIP | Full PSP | Infants and toddlers and term newborn | No significant therapeutic benefit^c^ | - |

^a^ The waiver reason in the EU: *”The medicinal product is likely to be ineffective or unsafe in part or all of the pediatric population”* or in the US: *”The medicinal product would be unsafe or ineffective for some or all pediatric age groups”*. ^b^ The waiver reason in the EU: *“The condition or disease for which the specific medicinal product or class is intended occurs only in the adult population”*. ^c^ The waiver reason in the EU: *“The specific medicinal product does not represent a significant therapeutic benefit over existing treatments for pediatric patients”*. ^d^The waiver reason in the US: *“The necessary studies are impossible or highly impracticable”*. ^e^ The waiver reason in the US: *“The medicinal product: Does not represent a meaningful therapeutic benefit over existing therapies for pediatric patients or Is not likely to be used in a substantial number of pediatric patients”.*

**Table S6: Comparison of paediatric requirements for indications granted at the time of initial approval of novel drugs in the EU and the US or indications only targeted by a PIP in the EU (n=284).**

|  | **Paediatric development plan** | | **No paediatric development** | | **P-value*** |
| --- | --- | --- | --- | --- | --- |
|  | **EU** | **US** | **EU** | **US** |  |
| **Adolescents** | 189 | 89 | 95 | 195 | <0.0001 |
| **Children** | 121 | 40 | 163 | 244 | <0.0001 |
| **Toddlers and infants** | 73 | 29 | 211 | 255 | <0.0001 |
| **Term newborn** | 65 | 25 | 219 | 259 | <0.0001 |

* Pearson's Chi-squared test with Yates' continuity correction

**Table S7: Comparison of paediatric requirements for indications granted at the time of initial approval of novel drugs in the EU and the US and being subject to the mandatory paediatric legislations in the EU and the US (n=131).**

|  | **Paediatric development plan** | | **No paediatric development** | | **P-value*** |
| --- | --- | --- | --- | --- | --- |
|  | **EU** | **US** | **EU** | **US** |  |
| **Adolescents** | 94 | 88 | 37 | 43 | 0.50 |
| **Children** | 45 | 39 | 86 | 92 | 0.51 |
| **Toddlers and infants** | 32 | 29 | 99 | 102 | 0.77 |
| **Term newborn** | 28 | 25 | 103 | 106 | 0.76 |

* Pearson's Chi-squared test with Yates' continuity correction
